#  The challenge of feedback-insights from non-medical educational research 

**DOI:** 10.5116/ijme.5489.eacb

**Published:** 2015-01-02

**Authors:** John Sandars

**Affiliations:** Medical Education Unit, The Medical School, The University of Sheffield, UK

I am frequently surprised that few medical educators appear to make use of the extensive educational research that has been performed in non-medical education settings. I can understand some of the scepticism, especially since some of the research has been performed in schools, but I consider that medical educators lose a potentially useful resource to help in their decision-making about how to implement educational interventions.

All research requires application to the particular context of the user, and this equally applies to educational research performed in medical and non-medical contexts. There are likely to be many differences between the context in which the research was initially performed and the setting in which the medical educator wishes to apply the findings from the research, For example, there may be differences in the phase of the curriculum, the previous experience of the students or the skills of the teacher delivering the intervention. At all times, the medical educator has to make a judgment and I consider that educational research that has been performed in non-medical education settings can be a useful aspect of making these judgments.

I will illustrate how insights from educational research about feedback in non-medical education contexts can be useful to medical educators. Feedback is currently a major challenge to medical educators. All final year students, including medical students, at a University in the United Kingdom are asked to complete the National Student Survey and their evaluation of the teaching that they have received consistently highlights that feedback is an area for improvement. In my experience, similar findings are also frequently noted when medical students are asked to evaluate both their academic and clinical teaching. Medical educators have to make decisions about how to provide effective feedback that can improve performance of students but there is still little research in medical education that can provide guidance.

## Feedback should be part of a wider assessment for learning approach

Many medical educators will be aware of "formative assessment" but my experience suggests that often this is separated from "feedback". Assessment for learning, often called formative assessment, has the priority intention of improving performance by making an assessment of how a student performs on a task and then providing feedback to the student about how the performance can be improved.^1^ This realisation that feedback is only one component of the wider approach of assessment for learning is important since there is a large research literature from non-medical settings that can inform how to make feedback more effective.

A review of 21 studies noted that all of the assessment for learning interventions, with students from school to undergraduate, improved performance.^2^ There was a mean effect size in each study between 0.4 and 0.7. The findings were consistent across different disciplines and countries. Interestingly, students who were "low attainers" appeared to improve the most.

The review highlighted that the assessment for learning interventions not only provided feedback to students but also allowed their teachers to modify their approach to teaching. There was also greater involvement of the students in the process, such as recognising the importance of assessments to improve their performance, instead of another hurdle to get over.

The insights from the review were subsequently used for a staff development programme in which teachers collaboratively developed action plans to improve their approach to assessment for learning.^3^ Examples of the interventions included improving awareness raising questions during teaching, providing clear feedback related to marking criteria and developing students to self-assess their own performance. The mean effect size of this intervention was 0.34.

The findings from this research have made an important impression on my thoughts about how feedback should be provided in medical education. For me, the findings highlight the need for both students and teachers to consider that feedback is only one aspect of a wider educational approach of assessment for learning, and that teachers need to adapt their teaching to ensure that students can achieve their potential.

## Appreciating the feedback preferences of students is important

My experience is that medical educators often assume how students prefer to be given feedback, without really understanding these preferences. There is medical educational research that shows what medical students dislike about how they have received feedback but there is little about what they prefer.

A large semi-structured questionnaire of 566 undergraduate and postgraduate students from Australia showed that there was a preference for written feedback which as positive and with clear comments that were linked to how the performance did, or did not, meet the assessment criteria and also how the performance could be improved to achieve these criteria.^4^ Students preferred feedback soon after the assessment but were willing to wait longer if the feedback was more detailed.

The findings from this research are not surprising to me, with students wanting detailed information about why they have not achieved the required standard and what they need to do to achieve the standard. These findings resonate with my experience and suggest that most students become extremely focussed about passing examinations, often adopting a superficial approach to learning that is limited to the assessment. This is a challenge to all medical educators since it is important for students to develop deeper learning but students often say to me that they do not really understand why they have not passed an assessment and what they can do to pass. The finding about wanting "positive" comments is interesting since students considered that this was essential to maintain motivation.

My reflection on this research suggests that teachers can respond by not merely providing specific answers for the questions but to direct the students to revisit their learning, including the use of different sources of information or to seek understanding of difficult concepts from their teachers. Providing encouraging "positive" comments is a timely reminder that, as medical educators, we are supporting our students to achieve their potential. However, it is essential that teachers balance their praise since it needs to be directed at the factors that students can control, such as effort or understanding of the task. Providing general encouragement that is not focussed can make students confused, especially students that are struggling. This comment is supported by a meta-analysis of over 23,000 observations from non-medical education contexts.^5^

## Use effective feedback approaches

There has been a review of 12 previous reviews of feedback effectiveness from non-medical education settings and there is a clear message that feedback to effectively improve performance requires careful provision of feedback that considers four components.^6^ The first component considers the need for providing "positive" feedback to maintain motivation and the second component is feedback on the extent to which a task was adequately performed or not. The third component considers what processes were used, or not used, to achieve performance of the task. The fourth, and most important aspect, is the self-regulation approach of the student as they perform the task. Self-regulation is a metacognitive ("thinking about thinking") process that coordinates the use of strategies and techniques to perform the task, such as goal setting, self-monitoring to ensure that the chosen strategies are working and adaptive change to make changes to ensure that the most appropriate strategies are being used.

In my experience, medical educators tend to avoid the essential self-regulation component of feedback but this is essential for students for the transfer of skills to other situations. A recent review of the medical education literature on the approaches for remediation of underperformance highlighted the need to provide a self-regulation component to the remediation package if long-term benefits were to be obtained, otherwise there was a tendency to only help students to get over the next assessment hurdle. ^7^

## Conclusion

I hope that this article has stimulated the reader to consider using educational research from non-medical education settings to inform their practice. I have found that this research has provided a welcomed fresh insight into the difficulties that I have experienced as a medical educator, especially in my understanding of how to make feedback more effective. An important aspect of the scholarship of being a medical educator is not only to use research to inform practice but to also research our own practice. I consider that the scepticism to using educational research from non-medical education settings can be overcome by practitioner research, in which there is a cyclical process of identification of problems, developing and implementing possible solutions, evaluating the impact of the solutions on the problem and refining the solutions to achieve a more complete resolution of the problem. Practitioner research accepts that professional practice is complex and social, with no clear solutions to problems. The cyclical process provides an opportunity to fit solutions to problems within a particular context. Insights from educational research from non-medical education settings can be used to develop possible solutions and we may be surprised to find that they fit the medical education context. I have certainly been surprised and I encourage medical educators to also adopt this approach.

## 

### Conflict of Interest

The author declares that there are no conflicts of interest.
